# Aberrant Cytokeratin 20 mRNA Expression in Peripheral Blood and Lymph Nodes Indicates Micrometastasis and Poor Prognosis in Patients With Gastric Carcinoma

**DOI:** 10.1177/1533033819832856

**Published:** 2019-03-03

**Authors:** Xiaolan You, Yuanjie Wang, Jian Wu, Qinghong Liu, Dehu Chen, Dong Tang, Daorong Wang

**Affiliations:** 1 Department of Gastrointestinal Surgery, The Hospital Affiliated to Medical School of Yangzhou University (Taizhou People's Hospital), Taizhou, Jiangsu Province, China; 2 Department of Gastrointestinal Surgery, Clinical Medical College of Yangzhou University, Subei People’s Hospital of Jiangsu Province, Yangzhou Jiangsu Province, China

**Keywords:** gastric cancer, cytokeratin, micrometastasis, prognosis, lymph nodes, peripheral blood

## Abstract

Several studies suggest that peripheral blood and lymph node micrometastases may be a causative factor for gastric cancer recurrence. Cytokeratin 20 shows enriched expression in intestinal epithelial cells. This study aimed to evaluate the clinical utility of monitoring cytokeratin 20 levels in peripheral blood and lymph nodes of patients with gastric cancer for detecting micrometastasis and predicting prognosis. We detected messenger RNA levels of cytokeratin 20 in gastric cancer cell lines and in the peripheral blood of 125 patients (85 patients with gastric cancer and 40 patients with benign neoplasm) by fluorescence quantitative real-time polymerase chain reaction both before and after radical resection. In all, 1586 lymph node samples from 85 patients with gastric cancer were evaluated for cytokeratin 20 expression using real-time polymerase chain reaction, as well as by immunohistochemistry staining with anti-pan-keratin and anti-cytokeratin 20 antibodies. All patients underwent follow-up until cancer-related death or for more than 3 years after tumor resection. We found that elevated cytokeratin 20 expression in peripheral blood as detected by quantitative real-time polymerase chain reaction closely correlates with poor clinicopathological characteristics. Detecting cytokeratin 20 messenger RNA in the lymph nodes by quantitative real-time polymerase chain reaction enabled more accurate determination of the clinicopathological staging of gastric cancer, best treatment approach, and prognosis. Our findings show that patients with increased cytokeratin 20 messenger RNA expression in the peripheral blood or lymph nodes have a shorter time to recurrence and poorer overall survival.

## Introduction

Gastric cancer (GC) is the fifth most common cancer worldwide and the third leading cause of cancer death in both sexes.^1,2^ Despite curative gastrectomy, extended lymph node dissection,^3-6^ and adjuvant chemotherapy, GC can recur in both regional and distant sites in the majority of patients, including those without lymph node metastasis based on conventional histological hematoxylin and eosin (H&E) staining.^7-9^ Several studies have concluded that peripheral blood and lymph node micrometastasis might be a key causative factor for GC recurrence.^10-14^

Lymph node micrometastases of 0.2 to 2.0 mm in size have been detected in lymph nodes by immunohistochemical (IHC)-based evaluation of cytokeratin (CK) expression, a component of the cell skeleton involved in cytomorphology.^15^ More recently, fluorescence quantitative real-time polymerase chain reaction (qRT-PCR) technology (which is a sensitive, specific, and rapid method) has been widely used to detect the presence of circulating cancer cells in the peripheral blood and lymph nodes.^16^ However, there are few reports on the detection and clinical significance of pre- and postoperative CK20 messenger RNA (mRNA) levels in the peripheral blood of patients with GC. Furthermore, the clinical impact of peripheral blood and lymph node micrometastasis on GC prognosis remains controversial.^17-19^ Several studies report that peripheral blood and lymph node micrometastasis in GC is a strong indicator of overt metastatic disease or poor prognosis.^20,21^ However, others have reported no significant correlation between micrometastasis and other clinic pathological characteristics and that the presence of micrometastasis does not influence patient prognosis.^22-26^

In the present study, we detected pre- and postoperative CK20 mRNA levels in the peripheral blood of patients with GC using qRT-PCR. We also detected CK20 expression in lymph node samples from 85 patients with GC using qRT-PCR as well as by IHC staining with anti-pan-keratin and anti-CK20 antibodies and estimated the clinical and prognostic value of these biomarkers in patients with GC.

## Materials and Methods

### Cell Lines and Culture Conditions

The GC lines AGS (moderately differentiated), SGC-7901 (moderately differentiated), N87 (well differentiated), BGC-823 (poorly differentiated), and MKN-45 (poorly differentiated) were purchased from the Type Culture Collection of the Chinese Academy of Sciences (Shanghai, China) and maintained with Roswell Park Memorial Institute medium (RPMI HyClone, Logan, Utah) supplemented with 10% fetal bovine serum (Thermo Scientific HyClone, Logan, Utah). All cell lines were maintained at 37°C in a humidified atmosphere containing 5% CO_2_.

### Patients

From January 2013 to June 2013, a total of 85 patients with GC who underwent gastrectomy at the Department of General Surgery of People’s Hospital of Taizhou City were enrolled in this study. The inclusion criteria of the study were as follows: (1) radical gastrectomy for the primary tumor and D2 lymphadenectomy following the Japanese Research Society for Gastric Cancer guidelines,^27^ (2) complete clinicopathological and follow-up data, (3) no prior chemotherapy or radiotherapy before surgery, (4) no gross or microscopic residual or recurrent gastric tumor, (5) no distant metastases prior to surgery, and (6) no other synchronous malignancies or serious disease in other systems.

The histological diagnosis and tumor–node–metastasis (TNM) staging were based on the seventh edition of the American Joint Committee on Cancer (AJCC) TNM staging system.^28^ The study protocol was approved by the Medical Ethics Committee of Taizhou People’s Hospital of Jiangsu Province, the Hospital Affiliated to Medical of Yangzhou University (TZRY-EC-13-0167), and all patients provided written informed consent.

Patient clinicopathologic parameters were collected, including gender, age, tumor location, tumor diameter, histological grade, Borrmann type, invasive depth, lymph node status, peripheral blood concentrations of cancer-related antigens (ie, carcino-embryonic antigen(CEA), carbohydrate antigen(CA)724, and CA199), and tumor emboli in the microvessels. A total of 40 patients with benign neoplasm were used as negative controls.

### Specimens

Before and after surgical resection, a 10 mL sample of peripheral blood was obtained from each patient through a catheter inserted into a peripheral vessel and collected into EDTA tubes. The blood samples were anticoagulated with heparin (5 U/mL), stored at 4°C, and processed within 4 hours after blood collection.

Half of each resected lymph node was fixed in 10% formalin and embedded in paraffin for routine histopathological and IHC examination. The other half was stored in a clean tube at −80°C for RNA extraction.

### Total RNA Isolation and Complementary DNA Synthesis

Mononuclear cells were isolated from 5 mL heparinized whole blood by density gradient centrifugation using a lymphocyte separating medium (Huajing Corp, Shanghai, China). Mononuclear cells and GC cell lines were washed 3 times with phosphate-buffered saline, then centrifuged at 2000 rpm for 5 minutes. Total RNA was then extracted from cell lysates using TRIzol reagent (Life Technologies, Gaithersburg, Maryland), according to the manufacturer’s instructions. For the lymph node samples, 50 to 100 mg of tissues were homogenized in 1 mL of TRIzol reagent using a power homogenizer prior to RNA extraction. The concentration and purity of RNA was determined using a NanoDrop spectrophotometer (Thermo Scientific, Wilmington, Delaware): A260 to A280 ratios in the range of 1.8 to 2.0 were considered pure. First-strand complementary DNA (cDNA) was synthesized using the SuperScript First-Strand cDNA Synthesis Kit (Invitrogen, Carlsbad, California), according to the manufacturer’s instructions, and then stored at −20°C for subsequent qPCR experiments.

### Fluorescence qRT-PCR

The mRNA expression levels of CK20 were detected by qRT-PCR using the ABI 7700 sequence detector (Perkin Elmer/Applied Biosystems, Foster City, California). β-Actin was used as an internal control to normalize mRNA levels. The primers targeting CK20 and β-actin were as follows: CK20 upstream primer: 5′-CAGACACACGGTGAACTATGG-3′ and downstream primer: 5′-GATCAGCTTCCACTGTTAGACG-3′; β-actin upstream primer: 5′-CACGAAACTACCTTCAACTCC-3′ and downstream primer: 5′-CATACTCCTGCTTGCTGATC-3′. All primers were synthesized by Life Technologies (Shanghai, China).

The amplification reaction mixture consisted of 4 μL cDNA, 25 μL 10× buffer, 1 μL dNTP, 0.4 μL Taq DNA polymerase, 1 μL each of sense and antisense primers (including CK20 and β-actin), and distilled H_2_O to a final volume of 50 μL. The amplification reaction mixture was amplified through 40 cycles. The amplification reaction mixture was first heated at 95°C for 5 minutes to terminate the reverse transcription reaction, and thermal cycling was carried out at 95°C for 30 seconds, 62°C for 30 seconds, and 72°C for 30 seconds, followed by final extension at 72°C for 10 minutes. The sample was then slowly cooled to 4°C. All reactions were run in triplicate. The same procedure was performed with the standard agent and a positive or negative control. The relative levels of normalized gene expression were calculated with the equation 2^−ΔΔCT^, in which ΔCT = CT gene − CT control. Receiver operating characteristic (ROC) statistics were employed to estimate the cut points to distinguish between high and low expression of CK20 in peripheral blood and lymph nodes from GC samples. The cutoff values for CK20 were determined in the blood samples of healthy donors and GC tissue using the maximal χ^2^ method.

### Immunohistochemistry

The IHC was performed as described previously,^29^ using anti-CK20 monoclonal antibody (Abcam, Cambridge, United Kingdom) and primary pan-CK antibody A45-B/B3 detecting CK8, CK18, and CK19 (AS Diagnostik, Hückeswagen, Germany). Negative controls were treated identically, though the primary antibodies were omitted, and positive controls were provided by the kit supplier. The results were assessed by 2 independent pathologists without knowledge of the patient clinical status. Positive staining was defined as moderate or strong brown or dark brown staining in cells. No visible staining or light brown staining in cells was defined as negative; detection of at least 0.2 mm foci combined with amplification H&E staining to confirm the configuration of cells was regarded as a positive case.

### Follow-Up

After curative resection, all patients were followed regularly every 3 months during the first 2 years and every 6 months during the third to fifth years. The final follow-up date was June 30, 2016. No patients were lost to follow-up. The median follow-up duration was 39.2 months (range: 36-43 months) after surgery. A total of 36 (42.4%) cases were metastasis or recurrence, of which 29 (34.1%) had tumor-related deaths.

### Statistical Analysis

Statistical analyses were performed with SPSS 16.0 for Windows (SPSS, Chicago, Illinois). The correlations between CK20 mRNA expression and clinicopathological features were analyzed using the χ^2^ test and the correlation of the 3 methods (H&E staining, IHC, and qRT-PCR) was calculated. Univariate analysis was performed to identify variables associated with prognosis. Cox regression analysis was used to identify independent predictors of prognosis in patients with GC. The survival rate curves were compared using the log-rank test. *P* values <.05 were considered statistically significant.

## Results

### Cytokeratin 20 mRNA Expressed in GC Cell Lines

**Figure 1.**
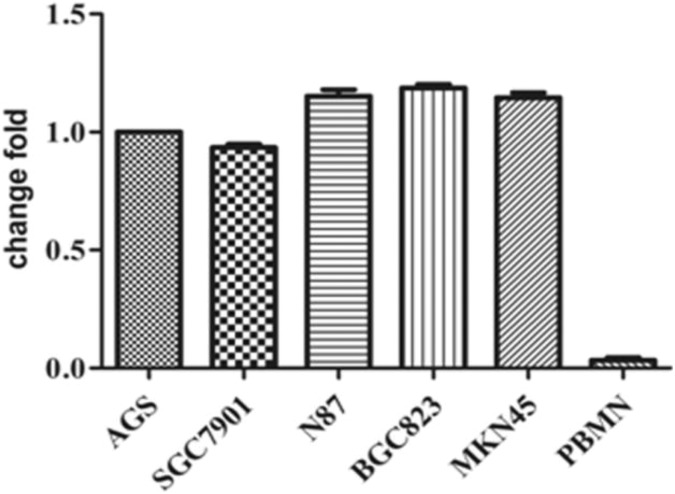
CK20 mRNA is expressed in multiple GC cell lines. There was no significant difference in CK20 expression among the 5 cell lines examined (*t* = 1.734, *P* = .181). CK20 indicates cytokeratin 20; mRNA, messenger RNA.

The CK20 mRNA was found to be expressed in GC cell lines, and there was no significant difference in CK20 expression among the 5 cell lines examined (*t* = 1.734, *P* = .181; Figure 1). Cell line SGC-7901 with the lowest expression of CK20 was selected for subsequent *in vitro* experiments. To validate the sensitivity of the qRT-PCR, different counts of SGC-7901 cells (10^5^, 10^4^, 10^3^, 10^2^, 10^1^, and 1) were added to a suspension containing 10^8^ peripheral blood mononuclear cells (PBMNs), and corresponding mRNA levels of CK20 were evaluated. We found the sensitivity for CK20 detection to be 10^−7^ in this *in vitro* model of gastric micrometastases.

### Patients and Characteristics

A total of 85 patients with GC were included in our study. There were 26 females (30.6%) and 59 males (69.4%), and the median age was 65.0 years (range: 35-80 years). All patients were histologically confirmed to have adenocarcinoma following pathological assessment of resected tumors. Participant characteristics and baseline measures are shown in Table 1. A total of 40 patients with benign tumors were used as negative controls, including 4 cases of gastric stromal tumor, 11 cases of thyroid adenoma, 7 cases of nodular goiter, 10 cases of benign breast tumor, and 8 cases of uterine fibroids.

**Table 1.** Correlations Between Preoperative CK20 mRNA Expression in Peripheral Blood and Clinicopathological Features of 85 Patients With Gastric Cancer.

Clinicopathological Feature	Negative, n (%)	Positive, n (%)	χ^2^ Test	*P* Value
	50 Cases (58.8%)	35 Cases (41.2%)		
Gender			0.666	.415
Male	33 (66.0)	26 (74.3)		
Female	17 (34.0)	9 (25.7)		
Age (years)			0.387	.543
≥60	40 (80.0)	26 (74.3)		
<60	10 (20.0)	9 (25.7)		
Tumor location			2.219	.528
Upper	16 (32.0)	16 (45.7)		
Middle	15 (30.0)	8 (22.9)		
Lower	16 (32.0)	8 (22.9)		
Diffuse/multiple lesions	3 (6.0)	3 (8.5)		
Tumor diameter (cm)			22.012	1.661 × 10^−5^
<3	24 (48.0)	1 (2.9)		
3-5	12 (24.0)	10 (28.6)		
>5	14 (28.0)	24 (68.5)		
Histological grade			5.873	.015
Well/moderately differentiated	26 (52.0)	9 (25.7)		
Poorly differentiated/undifferentiated	24 (48.0)	26 (74.3)		
Borrmann type			30.506	1.079 × 10^−6^
I	5 (10.0)	0 (0.0)		
II	26 (52.0)	3 (8.6)		
III	12 (24.0)	9 (25.7)		
IV	7 (14.0)	23 (65.7)		
Invasive depth			18.286	1.901 × 10^−5^
T1-T2	32 (64.0)	6 (17.1)		
T3-T4	18 (36.0)	29 (82.9)		
Lymph node status			58.564	1.968 × 10^−14^
N0	43 (86.0)	0 (0.0)		
N1	51 (0.0)	5 (14.3)		
N2	2 (4.0)	10 (28.6)		
N3	0 (0.0)	20 (57.1)		
CEA			30.310	3.683 × 10^−8^
Normal	48 (96.0)	15 (42.9)		
High	2 (4.0)	20 (57.1)		
CA724			29.628	5.234 × 10^−8^
Normal	47 (94.0)	14 (40.0)		
High	3 (6.0)	21 (60.0)		
CA199			25.739	3.908 × 10^−7^
Normal	48 (96.0)	17 (48.6)		
High	2 (4.0)	18 (51.4)		
Tumor emboli in the microvessels			27.811	1.338 × 10^−7^
Negative	33 (66.0)	3 (8.6)		
Positive	17 (34.0)	32 (91.4)		

Abbreviations: CA, carbohydrate antigen; CEA, carcino-embryonic antigen; CK20, cytokeratin 20; mRNA, messenger RNA.

### Correlation Between Preoperative CK20 mRNA Levels and Clinicopathological Factors

**Figure 2.**
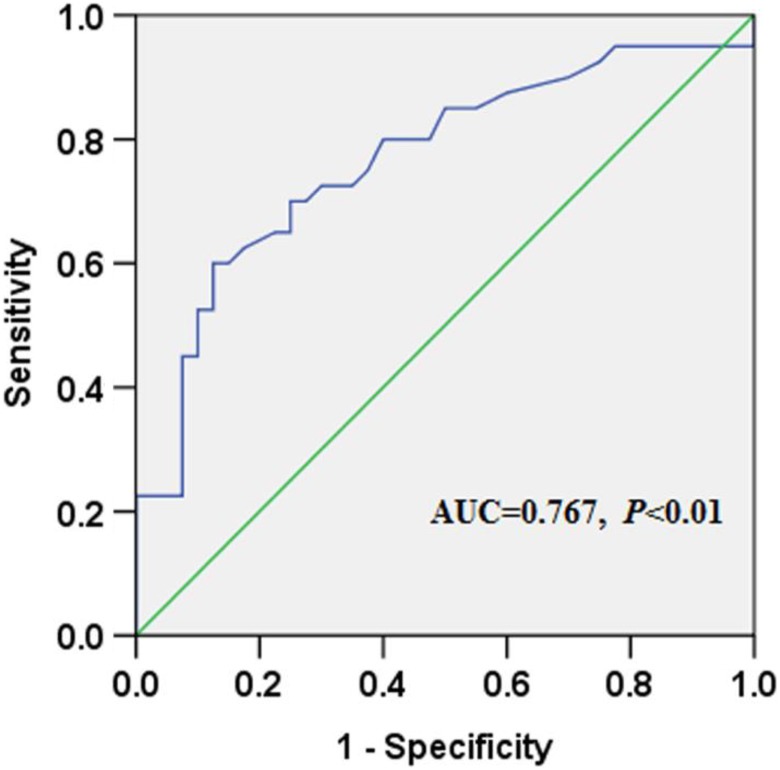
The ROC statistics were used to estimate the cutoff scores to distinguish between positive and negative CK20 mRNA expression. CK20 indicates cytokeratin 20; mRNA, messenger RNA; ROC, receiver operating characteristic.

In the blood samples of healthy donors, the median CK20 mRNA level was 0.43 (range: 0.27-2.15) and the median CK20 level in GC tissues was 0.93 (range: 0.23-4.17). The ROC statistics were used to estimate the cutoff scores to distinguish positive and negative CK20 expression. Scores ≥0.83 were considered to indicate positive expression (Figure 2). Based on these results, none of the negative controls showed positive CK20 expression preoperatively, while 35 of 85 patients with primary GC showed positive CK20 expression (positive ratio: 41.2%). A significant difference between the 2 groups was found (χ^2^ = 22.876, *P* = 1.72 × 10^−6^). The associations between the pre-CK20 mRNA levels and the clinicopathological characteristics are shown in Table 1.

Preoperative expression levels of CK20 in peripheral blood from patients with GC correlated with tumor diameter (*P* = 1.661 × 10^−5^), invasive depth (*P* = 1.901 × 10^−5^), differentiation grade (*P* = .015), Borrmann type (*P* = 1.079 × 10^−6^), lymphatic metastasis (*P* = 1.968 × 10^−14^), tumor emboli in the microvessels (*P* = 1.338 × 10^−7^), and concentration of cancer-related antigens in peripheral blood, including CEA (*P* = 3.683 × 10^−8^), CA724 (*P* = 5.234 × 10^−8^), and CA199 (*P* = 3.908 × 10^−7^). However, expression of CK20 in peripheral blood did not correlate with the demographic characteristics or tumor location (*P* > .05).

### Correlation Between Postoperative CK20 mRNA Levels and Clinicopathological Factors

On the first day after resection, CK20 levels as detected by qPCR were still negative among the 40 patients with benign tumors. In contrast, 49 cases (57.6%) in the patient with GC group showed positive CK20 expression. There was a significant difference between the malignant and benign tumor groups (χ^2^ = 37.926, *P* = 7.34 × 10^−10^). The associations between the post-CK20 mRNA levels and the clinicopathological characteristics are shown in Table 2.

**Table 2.** Correlations Between Postoperative CK20 mRNA Expression in Peripheral Blood and Clinicopathological Features of 85 Patients With Gastric Cancer.

Clinicopathological Feature	Negative, n (%)	Positive, n (%)	χ^2^ Test	*P* Value
	36 Cases (42.4%)	49 Cases (57.6%)		
Gender			0.232	.630
Male	26 (72.2)	33 (67.3)		
Female	10 (27.8)	16 (32.7)		
Age (years)			4.547	.053
≥60	32 (88.9)	34 (69.4)		
<60	4 (11.1)	15 (30.6)		
Tumor location			3.655	.301
Upper	10 (27.8)	22 (44.9)		
Middle	13 (36.1)	10 (20.4)		
Lower	10 (27.8)	14 (28.6)		
Diffuse/multiple lesions	3 (8.3)	3 (6.1)		
Tumor diameter (cm)			32.221	8.271 × 10^−9^
<3	23 (63.9)	2 (4.1)		
3-5	7 (19.4)	15 (30.6)		
>5	6 (16.7)	32 (65.3)		
Histological grade			3.470	.062
Well/moderately differentiated	19 (52.8)	16 (32.7)		
Poorly differentiated/undifferentiated	17 (47.2)	33 (67.3)		
Borrmann type			20.178	1.559 × 10^−4^
I	3 (8.3)	2 (4.1)		
II	21 (58.4)	8 (16.3)		
III	7 (19.4)	14 (28.6)		
IV	5 (13.9)	25 (51.0)		
Invasive depth			32.468	1.212 × 10^−8^
T1-T2	29 (80.6)	9 (18.4)		
T3-T4	7 (19.4)	40 (81.6)		
Lymph node status			48.412	1.739 × 10^−10^
N0	34 (94.4)	9 (18.4)		
N1	1 (2.8)	9 (18.4)		
N2	1 (2.8)	11 (22.4)		
N3	0 (0.0)	20 (40.8)		
CEA			17.378	3.064 × 10^−5^
Normal	35 (97.2)	28 (57.1)		
High	1 (2.8)	21 (42.9)		
CA724			19.974	7.852 × 10^−6^
Normal	35 (97.2)	26 (53.1)		
High	1 (2.8)	23 (46.9)		
CA199			19.215	2.805 × 10^−6^
Normal	36 (100.0)	29 (59.2)		
High	0 (0.0)	20 (40.8)		
Tumor emboli in the microvessels			42.955	5.600 × 10^−11^
Negative	30 (83.3)	6 (12.2)		
Positive	6 (16.7)	43 (87.8)		

Abbreviations: CA, carbohydrate antigen; CEA, carcino-embryonic antigen; CK20, cytokeratin 20; mRNA, messenger RNA.

Positive CK20 expression in peripheral blood postoperatively was closely related to tumor diameter (*P* = 8.271 × 10^−9^), Borrmann type (*P* = 1.559 × 10^−4^), invasive depth (*P* = 1.212 × 10^−8^), lymphatic metastasis (*P* = 1.739 × 10^−10^), microvessel invasion (*P* = 5.600 × 10^−11^), and cancer-related antigens, including CEA (*P* = 3.064 × 10^−5^), CA724 (*P* = 7.852 × 10^−6^), and CA199 (*P* = 2.805 × 10^−6^). Similar to findings for preoperative CK20 expression, there was no association between CK20 and demographic factors and tumor location (*P* > .05). Additionally, there was no association between CK20 and the degree of differentiation (*P* = .062).

### Cytokeratin 20 mRNA Levels in Postoperative Peripheral Blood Compared With Preoperative Levels

Among the 85 patients with GC, 35 (41.2%) showed positive CK20 expression in preoperative peripheral blood, while 49 (57.6%) had increased CK20 expression in postoperative peripheral blood. There was a significant increase in CK20 expression in postoperative peripheral blood compared with preoperative levels (χ^2^ = 4.612, *P* = .032).

We analyzed the clinicopathological features of patients with positive CK20 mRNA expression in postoperative blood alone compared to 50 patients with negative preoperative expression of CK20 mRNA. We found that expression of CK20 mRNA in postoperative blood alone was related to age (*P* = .012), tumor diameter (*P* = .001), invasive depth (*P* = .031), lymph node status (*P* = .016), and tumor emboli in the microvessels (*P* = 3.339 × 10^−5^). The associations between postoperative positive CK20 mRNA expression in peripheral blood alone and clinicopathological characteristics are shown in Table 3.

**Table 3.** Correlations Between Postoperative Positive CK20 mRNA Expression in Blood Alone and Clinicopathological Features of 50 Patients With Preoperative Negative CK20 mRNA Expression.

Clinicopathological Feature	Negative, n (%)	Positive, n (%)	χ^2^ Test	*P* Value
	36 Cases (72.0%)	14 Cases (28.0%)		
Gender			0.22	.187
Male	26 (72.2)	7 (50.0)		
Female	10 (27.8)	7 (50.0)		
Age (years)			6.34	.012
≥60	32 (88.9)	8 (57.1)		
<60	4 (11.1)	6 (42.9)		
Tumor location			4.20	.241
Upper	10 (27.8)	6 (42.9)		
Middle	13 (36.1)	2 (14.2)		
Lower	10 (27.8)	6 (42.9)		
Diffuse/multiple lesions	3 (8.3)	0 (6.1)		
Tumor diameter (cm)			13.77	.001
<3	23 (63.9)	1 (7.1)		
3-5	7 (19.4)	5 (35.7)		
>5	6 (16.7)	8 (57.2)		
Histological grade			0.031	.860
Well/moderately differentiated	19 (52.8)	7 (50.0)		
Poorly differentiated/undifferentiated	17 (47.2)	7 (50.0)		
Borrmann type			2.46	.482
I	3 (8.3)	2 (14.3)		
II	21 (58.4)	5 (35.7)		
III	7 (19.4)	5 (35.7)		
IV	5 (13.9)	2 (14.3)		
Invasive depth			4.66	.031
T1-T2	29 (80.6)	7 (50.0)		
T3-T4	7 (19.4)	7 (50.0)		
Lymph node status			8.25	.016
N0	34 (94.4)	9 (64.3)		
N1	1 (2.8)	4 (28.6)		
N2	1 (2.8)	1 (7.1)		
CEA			0.50	.479
Normal	35 (97.2)	13 (57.1)		
High	1 (2.8)	1 (42.9)		
CA724			0.50	.479
Normal	35 (97.2)	13 (53.1)		
High	1 (2.8)	1 (46.9)		
CA199			5.37	.074
Normal	36 (100.0)	12 (59.2)		
High	0 (0.0)	2 (40.8)		
Tumor emboli in the microvessels			17.25	3.339 × 10^−5^
Negative	30 (83.3)	3 (12.2)		
Positive	6 (16.7)	11 (87.8)		

Abbreviations: CA, carbohydrate antigen; CEA, carcino-embryonic antigen; CK20, cytokeratin 20; mRNA, messenger RNA.

### Correlation Between Peripheral Blood CK20 mRNA Levels and Patient Survival

**Figure 3.**
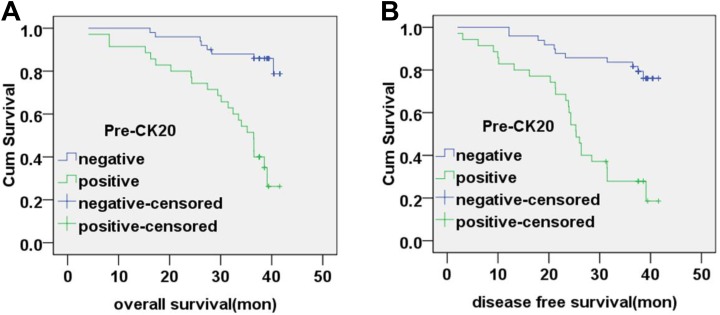
Correlation between preoperative CK20 mRNA expression and patient survival. A, The overall survival rate of patients based on preoperative CK20-negative and CK20-positive expression in peripheral blood (*P* < .01). B, The disease-free survival rate of patients based on preoperative CK20-negative and CK20-positive expression in peripheral blood (*P* < .01). CK20 indicates cytokeratin 20; mRNA, messenger RNA.

All patients underwent follow-up until cancer-related death or more than 3 years after tumor resection. The median follow-up interval was 39.2 months. The overall survival (OS) time was 39.20 ± 0.87 months (95% confidence interval [CI]: 37.51-40.90) for preoperative CK20-negative patients and 31.62 ± 1.84 months (95% CI: 28.01-35.23) for preoperative CK20-positive patients. The disease-free survival (DFS) time was 37.72 ± 1.19 months (95% CI: 35.38-40.05) for preoperative CK20-negative patients and 25.99 ± 2.01 months (95% CI: 22.06-29.03) for preoperative CK20-positive patients. The OS and DFS time for preoperative CK20-positive patients was significantly poorer than that of CK20-negative patients (χ^2^ = 24.13, *P* < .01; χ^2^ = 25.68, *P* < .01; respectively). Correlation between preoperative CK20 mRNA levels and patient survival are shown in Figure 3.

**Figure 4.**
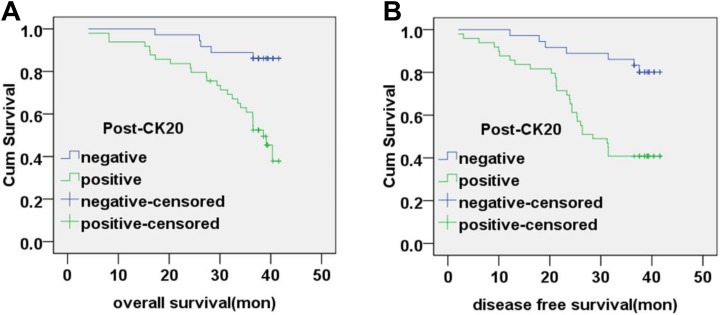
Correlation between postoperative CK20 mRNA expression and patient survival. A, The overall survival rate of patients based on postoperative CK20-negative and CK20-positive expression in peripheral blood (*P* < .01). B, The disease-free survival rate of patients based on postoperative CK20-negative and CK20-positive expression in peripheral blood (*P* < .01). CK20 indicates cytokeratin 20; mRNA, messenger RNA.

The OS time was 39.52 ± 0.93 months (95% CI, 37.69-41.35) for postoperative CK20-negative patients and 33.73 ± 1.50 months (95% CI: 30.77-36.69) for postoperative CK20-positive patients. The DFS time was 38.41 ± 1.26 months (95% CI: 35.94-40.87) for postoperative CK20-negative patients and 29.01 ± 1.76 months (95% CI, 25.57-32.46) for postoperative CK20-positive patients. The OS and DFS time for postoperative CK20-positive patients was significantly poorer than that of CK20-negative patients (χ^2^ = 13.613, *P* < .01; χ^2^ = 11.065, *P* < .01; respectively). Correlation between postoperative CK20 mRNA levels and patient survival is shown in Figure 4.

### Detection of CK20 Expression in Lymph Nodes by IHC and qRT-PCR

A total of 1568 lymph nodes were removed from 85 patients with GC, with a median of 18.5 (range: 7-90) lymph nodes per patient. By H&E staining, 414 lymph nodes and 42 patients were positive for metastasis, with a median of 4.8 (range: 1-89) lymph nodes per patient. Lymph nodes positive for metastasis by H&E staining were all positive via pan-CK IHC. For specimens detected by CK20-IHC, 44 (10.6%) lymph nodes positive for metastasis by H&E staining showed negative CK expression. All lymph nodes that were negative for metastasis by H&E staining were further evaluated for CK staining using pan-CK IHC. Among these, an additional 28 (2.43%) lymph nodes representing 16 patients showed positive pan-CK expression by IHC. When evaluated by qRT-PCR, an additional 137 (11.87%) lymph nodes representing 34 patients showed positive CK20 mRNA expression. All IHC-positive lymph nodes were confirmed as positive by qRT-PCR. The sensitivity of qRT-PCR was significantly higher than H&E staining and IHC in GC lymph nodes (χ^2^ = 31.87, *P* < .01), and there was a statistically significant difference between CK20 detection by RT-PCR compared with pan-CK detection by IHC in lymph nodes negative for metastasis by H&E staining (χ^2^ = 9.18, *P* = .002).

Based on the expression of CK20 mRNA, we identified 137 lymph nodes and 34 patients with micrometastases. This resulted in a change in clinicopathological staging in 22 cases. Correlation between the postoperative CK20 mRNA expression in lymph nodes and clinicopathological factors was closely related to the degree of differentiation (*P* = 3.19 × 10^−4^), invasive depth (*P* = 4.188 × 10^−5^), microvessel invasion (*P* = 1.673 × 10^−4^), the tumor diameter (*P* = .019), Borrmann type (*P* = .049), tumor-related antigens including CEA (*P* = .034), CA724 (*P* = .008), and CA199 (*P* = .037), as well as preoperative (*P* = .024) and postoperative CK20 expression in peripheral blood (*P* = .004). However, no association was observed between postoperative CK20 mRNA expression and demographic characteristics, tumor location, or the status of lymphatic metastasis (*P* > .05). The correlation between CK20 mRNA levels in lymph nodes and clinicopathological characteristics is shown in Table 4.

**Table 4.** Correlations Between CK20 mRNA Expression in Lymph Nodes and Clinicopathological Features of 85 Patients With Gastric Cancer.

Clinicopathological Feature	Negative, n (%)	Positive, n (%)	χ^2^ Test	*P* Value
	51 Cases (60.0%)	34 Cases (40.0%)		
Gender			1.561	.212
Male	38 (74.5)	21 (61.7)		
Female	13 (25.5)	13 (38.2)		
Age (years)			0.554	.457
≥60	41 (80.4)	25 (73.5)		
<60	10 (19.6)	9 (26.5)		
Tumor location			0.151	.985
Upper	19 (37.2)	13 (38.2)		
Middle	14 (27.5)	9 (26.5)		
Lower	14 (27.5)	10 (29.4)		
Diffuse/multiple lesions	4 (7.8)	2 (5.9)		
Tumor diameter (cm)			7.976	.019
<3	20 (39.2)	5 (14.7)		
3-5	14 (27.5)	8 (23.5)		
>5	17 (33.3)	21 (61.8)		
Histological grade			12.952	3.19 × 10^−4^
Well/moderately differentiated	29 (56.9)	6 (17.6)		
Poorly differentiated/undifferentiated	22 (43.1)	28 (82.4)		
Borrmann type			7.787	.049
I	5 (9.8)	0 (0.0)		
II	21 (41.2)	8 (23.5)		
III	10 (19.6)	11 (32.4)		
IV	15 (29.4)	15 (44.1)		
Lymph node status (H&E staining)			4.194	.241
N0	31 (60.8)	12 (35.3)		
N1	7 (13.7)	3 (8.8)		
N2	5 (9.8)	7 (20.6)		
N3	8 (15.7)	12 (35.3)		
Invasive depth			16.784	4.188 × 10^−5^
T1-T2	32 (62.7)	6 (17.6)		
T3-T4	19 (37.3)	28 (82.4)		
CEA			4.508	.034
Normal	42 (82.4)	21 (61.8)		
High	9 (17.6)	13 (38.2)		
CA724			7.054	.008
Normal	42 (82.4)	19 (55.9)		
High	9 (17.6)	15 (44.1)		
CA199			4.359	.037
Normal	42 (82.4)	23 (67.6)		
High	9 (17.6)	11 (32.4)		
Tumor emboli in the microvessels			14.167	1.673 × 10^−4^
Negative	30 (58.8)	6 (17.6)		
Positive	21 (41.2)	28 (82.4)		
Pre-CK20 in peripheral blood			5.006	.024
Negative	35 (68.6)	15 (44.1)		
Positive	16 (31.4)	19 (55.8)		
Post-CK20 in peripheral blood			8.224	.004
Negative	28 (54.9)	8 (23.5)		
Positive	23 (45.1)	26 (76.5)		

Abbreviations: CA, carbohydrate antigen; CEA, carcino-embryonic antigen; CK20, cytokeratin 20; H&E, hematoxylin and eosin; mRNA, messenger RNA.

### Correlation Between CK20 mRNA in Lymph Nodes and Patient Survival

The OS time was 38.76 ± 1.39 months (95% CI: 36.03-41.49) for CK20 mRNA-negative lymph node patients and 34.59 ± 1.32 months (95% CI: 31.99-37.16) for CK20 mRNA-positive lymph node patients. The OS time for CK20 mRNA-positive lymph node patients was significantly poorer than that of CK20 mRNA-negative lymph node patients (χ^2^ = 22.435, *P* < .01).

**Figure 5.**
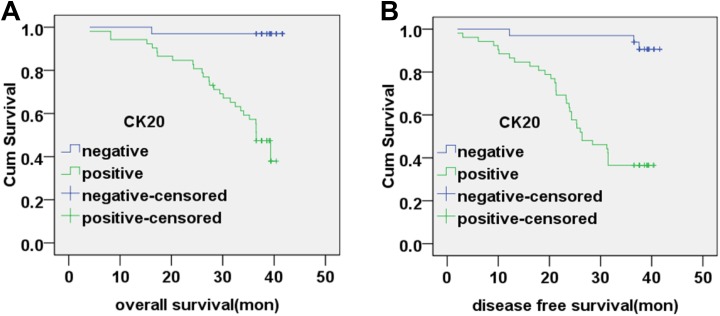
Correlation between CK20 mRNA expression in lymph nodes and patient survival. A, The overall survival rate of patients based on CK20-positive expression and CK20-negative expression in lymph nodes (*P* < .01). B, The disease-free survival rate of patients based on CK20-positive expression and CK20-negative expression in lymph nodes (*P* < .01). CK20 indicates cytokeratin 20; mRNA, messenger RNA.

The DFS time was 38.41 ± 1.26 months (95% CI, 38.64-42.13) for CK20 mRNA-negative lymph node patients and 29.01 ± 1.76 months (95% CI, 25.57-32.46) for CK20 mRNA-positive lymph node patients. The DFS time for CK20 mRNA-positive lymph node patients was significantly poorer than that of negative patients (χ^2^ = 24.042, *P* < .01). Correlation between the CK20 mRNA status in lymph nodes and patient survival is shown in Figure 5.

### Factors Associated With Survival of Patients With GC

On univariate analysis, tumor diameter, histological grade, Borrmann type, invasive depth, CEA, CA724, CA199, tumor emboli in the microvessels, preoperative CK20 in peripheral blood, postoperative CK20 in peripheral blood, and lymph node metastasis status by H&E staining, pan-CK expression by IHC, and CK20 mRNA expression by qPCR were significantly associated with survival (Table 5). On Cox regression analysis, histological grade, preoperative CK20 in peripheral blood, postoperative CK20 in peripheral blood, and lymph node status by H&E staining, pan-CK expression by IHC, and CK20 mRNA expression by qPCR were independently associated with survival of patients with GC (Table 6).

**Table 5.** Univariate Analysis Showing Variables Associated With Survival of Patients With Gastric Cancer.

Clinicopathological Feature	Died, n (%)	Survived, n (%)	χ^2^ Test	*P* Value
	29 Cases (34.1%)	56 Cases (65.9%)		
Gender			0.004	.95
Male	20 (69.0)	39 (69.6)		
Female	9 (31.0)	17 (30.4)		
Age (years)			3.73	.06
≥60	19 (65.5)	47 (83.9)		
<60	10 (34.5)	9 (16.1)		
Tumor location			3.72	.29
Upper	10 (34.5)	22 (39.3)		
Middle	6 (20.7)	17 (30.3)		
Lower	9 (31.0)	15 (26.8)		
Diffuse/multiple lesions	4 (13.8)	2 (3.6)		
Tumor diameter (cm)			13.69	.001
<3	4 (13.8)	21 (37.5)		
3-5	4 (13.8)	18 (32.1)		
>5	21 (72.4)	17 (30.4)		
Histological grade			21.35	3.81 × 10^−6^
Well/moderately differentiated	2 (6.9)	33 (58.9)		
Poorly differentiated/undifferentiated	27 (93.1)	23 (41.1)		
Borrmann type			10.34	.016
I	0 (0.0)	5 (8.9)		
II	5 (17.3)	24 (42.9)		
III	9 (31.0)	12 (21.4)		
IV	15 (51.7)	15 (26.8)		
Invasive depth			7.53	.006
T1-T2	7 (24.1)	31 (55.4)		
T3-T4	22 (75.9)	25 (44.6)		
CEA			8.23	.004
Normal	16 (55.2)	47 (83.9)		
High	13 (44.8)	9 (16.1)		
CA724			5.98	.014
Normal	16 (55.2)	45 (80.4)		
High	13 (44.8)	11 (19.6)		
CA199			7.79	.005
Normal	17 (58.6)	48 (85.7)		
High	12 (41.4)	8 (14.3)		
Tumor emboli in the microvessels			11.36	.001
Negative	5 (17.2)	31 (55.4)		
Positive	24 (82.8)	25 (44.6)		
Pre-CK20 in peripheral blood			17.73	2.54 × 10^−5^
Negative	8 (27.6)	42 (75.0)		
Positive	21 (72.4)	14 (25.0)		
Post-CK20 in peripheral blood			1.36	.001
Negative	5 (17.2)	31 (55.4)		
Positive	24 (82.8)	25 (44.6)		
Lymph node status (H&E staining)			19.58	9.63 × 10^−6^
Negative	5 (17.2)	38 (67.9)		
Positive	24 (82.8)	18 (32.1)		
Lymph node status (IHC pan-CK)			28.49	9.37 × 10^−8^
Negative	2 (6.9)	38 (67.9)		
Positive	27 (93.1)	18 (32.1)		
Lymph node status (PCR CK20)			18.89	1.38 × 10^−5^
Negative	2 (6.9)	31 (55.4)		
Positive	27 (93.1)	25 (44.6)		

Abbreviations: CA, carbohydrate antigen; CEA, carcino-embryonic antigen; CK20, cytokeratin 20; H&E, hematoxylin and eosin; IHC, immunocytochemistry; PCR, polymerase chain reaction.

**Table 6.** Cox Regression Analysis Showing Variables Independently Associated With Survival of Patients With Gastric Cancer.

	*B*	SE	Wald	Significance	Exp(B)	95% CI for Exp(B)	
						Lower	Upper
Histological grade	−1.87	0.61	9.29	0.002	0.156	0.05	0.51
Preoperative CK20 in peripheral blood	1.62	0.43	14.00	<0.001	5.04	2.16	11.77
Postoperative CK20 in peripheral blood	1.14	0.52	4.80	0.03	3.13	1.13	8.71
Lymph node status (H&E staining)	−1.52	0.64	5.58	0.02	0.22	0.06	0.77
Lymph node status (IHC pan-CK)	4.25	0.95	19.83	<0.001	70.01	10.79	454.25
Lymph node status (PCR CK-20)	1.87	0.76	6.10	0.01	6.48	1.47	28.57

Abbreviations: CA, carbohydrate antigen; CEA, carcino-embryonic antigen; CI, confidence interval; CK20, cytokeratin 20; H&E, hematoxylin and eosin; IHC, immunocytochemistry; PCR, polymerase chain reaction; SE, standard error.

## Discussion

Despite efforts regarding prevention, early diagnosis, and improved therapeutic strategies, mortality rates for patients with GC with resectable tumors remain high. Invasion and distant metastasis are the leading factors influencing the clinical outcome of patients with GC.^30^ Several studies have also shown that peripheral blood and lymph node micrometastasis prior to surgery might be key indicators for GC recurrence and distant metastasis.^17,31,32^ Therefore, identifying markers to determine the potential of metastasis and disease progression in patients with GC will help us tailor postoperative adjuvant therapies and improve clinical outcomes. However, currently, there are no markers available that can predict peripheral blood or lymph node metastasis or evaluate the prognosis of patients with GC.

Previous studies have shown that CK18 and CK19 mRNA could be detected in peripheral blood using nested RT-PCR in healthy participants, but CK20 mRNA could not be detected.^33^ Our results indicated that CK20 mRNA is expressed in different GC cell lines and that detection of CK20 using RT-PCR is a relatively sensitive and specific method to distinguish gastric cells from PBMNs. We also found that none of the patients with benign neoplasm showed positive CK20 expression in the peripheral blood before or after surgery. The CK20 was only expressed in the peripheral blood from patients with GC. Because CK20 is specifically found in gastrointestinal epithelium,^34,35^ our results indicate that epithelial cells or epithelial carcinoma cells exist in the peripheral blood circulation.

In our study, we detected 35 (41.2%) of 85 patients with primary GC with positive CK20 expression by qRT-PCR prior to surgical resection. In contrast, we detected 49 (57.6%) of 85 patients with positive CK20 expression by qRT-PCR on the first day after the operation. Postoperatively, there was no association between positive CK20 mRNA detection and histological grade (*P* > .05). Our findings indicate that micrometastasis in the peripheral blood is associated with surgery and that the operative procedure may be a factor in promoting micrometastasis. We also found that OS and DFS for patients with positive CK20 expression were significantly poorer than that of patients with negative CK20 expression (*P* < .01). This suggests that CK20 expression in the peripheral blood is indicative of micrometastasis and poor prognosis in patients with GC.

Currently, micrometastases in lymph nodes are defined as tumor cell clusters of between 0.2 and 2.0 mm in size in the greatest dimension. According to the seventh TNM classification by the International Union Against Cancer,^36^ lymph node micrometastasis should be reflected in the staging of the disease. The IHC is a widely accepted technique for detecting lymph node micrometastasis in GC using epithelial markers such as CK.^37^ Using IHC, Cai *et al* reported the incidence of lymph node micrometastasis was 25% in T1b GC,^38^ while Kim *et al* reported a 10% incidence of lymph node micrometastasis in pT1N0 GC.^24^ In our study, we found that 44 (10.6%) lymph nodes positive for micrometastasis based on H&E staining were negative for CK20 expression based on IHC, indicating that use of an anti-CK20 antibody to detect lymph node micrometastases may result in false negatives. However, all lymph nodes positive for micrometastasis based on H&E staining were also positive for pan-CK expression based on IHC, and the incidence of lymph node micrometastasis based on pan-CK IHC was 2.43% (28/1154) among the lymph nodes that were called as negative by H&E staining.

When micrometastasis is detected by RT-PCR, CK is usually employed as a target marker.^39,40^ The RT-PCR was previously shown to increase the detection rate of micrometastasis to a level higher than that achieved by IHC.^40^ According to the report by Arigami *et al*, RT-PCR identified lymph node micrometastasis in 31.3% of patients, whereas IHC detected lymph node micrometastasis in 11.3% of patients.^41^ Similarly, we found the sensitivity of qRT-PCR was significantly higher than IHC (*P* < .01).

According to the seventh edition of the AJCC staging system,^28^ if the number of macrometastatic nodes is less than 15, detection of only one micrometastasis could change the N stage. From our study in which the expression of CK20 mRNA was detected in lymph nodes, we identified 137 lymph nodes in 34 patients with micrometastases. Our findings changed the clinicopathological staging in 22 cases, and we found that micrometastasis in lymph nodes is significantly related to indicators of poor prognosis.

## Conclusions

This study showed that CK20 mRNA expression in the lymph nodes is indicative of lymph node micrometastasis. Until now, the clinical impact of lymph node micrometastasis on GC prognosis was controversial. Our results indicate that expression of CK20 mRNA in lymph nodes was significantly associated with reduced OS and DFS. Therefore, detecting CK20 mRNA expression in the peripheral blood and lymph nodes might help to diagnose micrometastasis in the circulation and thus identify patients with GC with poor prognosis. Indeed, micrometastasis is thought to be a key etiology of recurrence and distant metastasis after resection of primary gastric tumors. We suggest the status of micrometastases in the peripheral blood in patients with early or advanced GC should be investigated at an early stage, so their treatment plan can be adjusted accordingly. In addition, the detection of micrometastases in the lymph nodes can allow more accurate clinicopathological staging of GC to guide treatment and improve prognosis.

## Abbreviations

cDNA: complementary DNA
CA: carbohydrate antigen
CEA: carcino-embryonic antigen
CI: confidence interval
CK20: cytokeratin 20
DFS: disease-free survival
GC: gastric cancer
H&E: hematoxylin and eosin
IHC: immunocytochemistry
mRNA: messenger RNA
OR: odds ratio
OS: overall survival
PBMN: peripheral blood mononuclear cell
qRT-PCR: quantitative real-time polymerase chain reaction
ROC: receiver operating characteristic
TNM: tumor–node–metastasis
